# Dr. Benjamin W. J. Worrall

**Published:** 1917-11

**Authors:** 


**Dr. Benjamin W. J. Worrall,** University of Kansas 1906, Lt. U. S. N., was killed in an automobile accident at N. Tonawanda, Sept. 15.

